# Language Lateralization and Auditory Attention Impairment in Young Adults at Ultra-High Risk for Psychosis: A Dichotic Listening Study

**DOI:** 10.3389/fpsyg.2018.00608

**Published:** 2018-04-27

**Authors:** Ingvild Aase, Kristiina Kompus, Jens Gisselgård, Inge Joa, Jan O. Johannessen, Kolbjørn Brønnick

**Affiliations:** ^1^Centre for Clinical Research in Psychosis (TIPS), Stavanger University Hospital, Stavanger, Norway; ^2^Institute of Health, Faculty of Social Science, University of Stavanger, Stavanger, Norway; ^3^Department of Biological and Medical Psychology, University of Bergen, Bergen, Norway

**Keywords:** ultra-high risk for psychosis (UHR), at mental high-risk, auditory attention, language lateralization, dichotic listening, Structural Interview for prodromal symptoms *significant correlations*

## Abstract

**Objectives:** Impaired attention and language functions are common in psychosis, but have been less explored in subjects with ultra-high risk for psychosis (UHR). The aim of the study was to investigate differences in language lateralization and auditory attention in UHR subjects compared to healthy controls with a dichotic listening paradigm. In addition, symptoms from The Structural Interview for Prodromal Syndromes (SIPS) were explored in relation to performance on dichotic listening.

**Methods:** The UHR subjects (*n* = 46, female = 28, mean age = 17.9) were compared to a group of healthy controls (*n* = 40, female = 20, mean age = 16.8). A split-plot repeated measures analysis of covariance was conducted with group as between-subjects factor and attention conditions (non-forced, forced-right, forced-left) and side (right ear, left ear) as repeated measures factors (2×3×2 design) using gender, age and handedness as covariates. SIPS symptoms were subjected to Spearman’s *r* correlations with laterality indexes and attentional gain in each ear.

**Results:** There was a statistically significant three-way interaction of group (UHR, healthy controls) × forced condition (non-forced, forced-right, forced-left) × side (right ear, left ear), *p* = 0.048. The effect was due to an interaction between group × side in the forced-left condition. There were no significant differences between UHR subjects and healthy controls in the non-forced condition. Right ear gain correlated with “Perceptual abnormalities/Hallucinations” (P4), *r* = 0.486, *p* = 0.001.

**Conclusion:** UHR subjects demonstrated impairment in top-down attentional mechanisms, but showed no language lateralization abnormalities. Impairment in top-down attentional mechanisms are frequently reported from dichotic listening studies in patients with schizophrenia. Higher levels of perceptual abnormalities and hallucinatory experiences were associated with enhanced report from the right ear in the forced-right condition.

## Introduction

The psychosis high-risk state, or ultra-high risk state (UHR) has been increasingly acknowledged as a valid nosological entity (Fusar-Poli et al., 2013). UHR, psychosis and schizophrenia are mental states which appear on a continuum as outlined in the continuum model of psychosis (Johns and Van Os, 2001) and the “psychosis-proneness-persistence impairment model of psychosis” (van Os et al., 2009). The continuum model of psychosis implies that psychotic symptoms may vary in severity from mild and infrequent psychosis-like symptoms in otherwise healthy individuals to schizotypal traits, schizotypal personality disorder to a diagnosable primary psychotic disorder (Esterberg and Compton, 2009). Comprehensive descriptions of UHR symptoms are presented in Yung and McGorry (1996) and Fusar-Poli et al. (2013).

Cognitive abnormalities have been extensively documented in schizophrenia (Rund, 2002; Schaefer et al., 2013) and cognitive deficits are present also in the UHR population (Pukrop et al., 2006, 2007; Pukrop and Klosterkötter, 2010; Bora et al., 2014). Additionally, there is a growing body of research from neuroimaging studies finding structural (Pantelis et al., 2003; Mechelli et al., 2011) and functional brain changes (Pugliese et al., 2007; Fusar-Poli et al., 2012) in UHR.

The anatomical division between the two hemispheres along the longitudinal fissure is a distinct characteristic of the brain and the left hemisphere is specialized for language and speech perception (Davidson and Hugdahl, 1996; Lezak, 2004; Hugdahl and Westerhausen, 2010). It has been proposed that abnormal language lateralization may be an etiological factor in schizophrenia (Crow, 2000, 2004; Nygård et al., 2013). Further, schizophrenia has been referred to as a left hemisphere disorder, as core symptoms of schizophrenia can hardly be understood except within the framework of language (e.g., voices commenting, thought insertion, thought broadcast, primary delusions and hallucinations) (Crow, 2004). Auditory hallucinations have been proposed as internally generated speech percepts lateralized in the left Peri-Sylvian region (Hugdahl et al., 2007). Central auditory processing deficits have also been detected in first-episode psychosis (Iliadou et al., 2013). Ocklenburg et al. (2013) conducted two meta-analyses of dichotic listening studies on language lateralization and auditory hallucinations. The first meta-analysis compared patients with schizophrenia to healthy controls, and the other compared schizophrenia patients with auditory hallucinations to healthy controls. Patients with schizophrenia had weaker language lateralization than healthy controls, but the effect size was small (*g* = -0.26). When comparing schizophrenia patients with auditory hallucinations to healthy controls a marked reduction in language lateralization was revealed (*g* = -0.45).

Language is not only lateralized in the brain, but is also contingent on attentional mechanisms such as in speech perception when selectively focusing on certain sounds while excluding others from mental processing. The capacity of the brain to process incoming stimuli is limited, and attention can be categorized into bottom-up and top-down processes, mediated by different neural mechanisms. Bottom-up processing is externally driven and is observable as attentional capture effects, for instance if a stimulus “stands out” from the background. This phenomenon is also named stimulus saliency. Top-down attention is internally guided based on prior knowledge, rules, current goals and instructions (Katsuki and Constantinidis, 2014). Attention deficits are frequently reported in psychotic disorders (McGhie and Chapman, 1961; Rund, 1985; Braff, 1993; Egeland et al., 2003). Deficient aspects of attention demonstrated in schizophrenia are early information-processing (Braff, 1993), cognitive control (Falkenberg et al., 2011), executive control (Wang et al., 2005; Nygård et al., 2013) and top-down vs. bottom-up attention (Asbjornsen and Hugdahl, 1995; Hugdahl et al., 2009a). Attention deficits have also been found in UHR (Pukrop and Klosterkötter, 2010; Lin et al., 2011).

Dichotic listening enables the assessment of both language lateralization and language-related selective attention (Hugdahl and Andersson, 1986; Hugdahl et al., 2009a; Kompus et al., 2012; Løberg et al., 2015). In the procedure used in the referenced studies, dichotic listening is administered by presenting one consonant-vowel (CV) syllable to each ear simultaneously and then asking the subjects to either report the syllable heard most clearly (non-forced), or the syllable presented to the right (forced-right) or left (forced-left). In the general population, there is a tendency to report more consonant-vowel syllables from the right ear. This effect has been labeled the right ear advantage (REA) (Hugdahl and Andersson, 1986; Sætrevik, 2012). Since language and linguistic abilities are lateralized to the left hemisphere, the REA has been postulated as a valid measure of cerebral language asymmetry (Hugdahl and Andersson, 1986; Hirnstein et al., 2013). An extensive body of research has found the REA in all age groups, in both females and males and in both right- and left-handed individuals (Hugdahl, 2009).

The conditions in dichotic listening assesses different cognitive mechanisms. The non-forced condition mainly taps a lateralized perceptual language component and the two other conditions additionally recruits attentional mechanisms (Hugdahl et al., 2009a; Kompus et al., 2012).

In a study by Løberg et al. (1999), patients with schizophrenia reported fewer CV syllables from the right ear in the non-forced condition than healthy controls. This was interpreted as a sign of reduced language lateralization (Løberg et al., 1999). Schizophrenia patients also show reduced report of stimuli from the left ear in the forced left condition as compared to healthy controls (Løberg et al., 2004; Nygård et al., 2013). A decreased REA in dichotic listening studies have been reported in healthy parents of children with schizophrenia, and this provides evidence for a hypothesized genetic origin of decreased lateralization in schizophrenia (Sommer et al., 2001). Further, a decrease in the ability to report and process right ear stimuli is linked to the severity of hallucinations in patients with schizophrenia, implying that language lateralization differences are more profound in the patients with auditory hallucinations (Ocklenburg et al., 2013). Analysis of fMRI images during dichotic presentations of CV syllables have shown a reduced overall activation in the left temporal lobe and the anterior cingulate cortex in patients with schizophrenia with auditory hallucinations compared to healthy controls (Hugdahl et al., 2009a).

### Research Questions

The main research aim of this study was to investigate whether UHR subjects and healthy controls could be distinguished in terms of characteristics in auditory attention and language lateralization measured with dichotic listening. Hypothesis 1: We expected to find deficits in attention in the UHR subjects as compared to healthy controls, defined as reduced ability to selectively report syllables from the left ear in the forced left condition. Hypothesis 2: We expected to find less language lateralization in the UHR subjects as compared to healthy controls, defined as an attenuated REA in the non-forced condition.

The secondary research aim was to explore the relationship between UHR symptoms and language lateralization in addition to attentional mechanisms as measured by dichotic listening. Positive-, Negative-, Disorganized-, and Generalized symptom clusters were explored as related to dichotic listening performance. The UHR symptoms perceptual abnormalities/hallucinations were explored with regard to both language lateralization and attention. Finally, UHR symptoms were selected for further investigation due to their postulated specific relevance to the main research question: Disorganized communication and expression of emotion were explored with regard to language lateralization, trouble with focus and attention was explored with regard to the attentional functions measures by the forced left and forced right conditions.

## Materials and Methods

### Participants

The UHR patients and matched healthy controls were recruited from the Norwegian Prevention of Psychosis (POP) study (Joa et al., 2015). Patient inclusion and exclusion criteria were: (1) Listed in the national register of South Rogaland County; (2) 13–65 years; (3) Fulfilling the diagnostic criteria for prodromal syndrome- SIPS criteria (Miller et al., 2003); (4) No current or life-time criteria for any psychotic disorder; (5) The symptoms are not better accounted for by an axis 1, axis 2 or substance abuse disorder; (6) No antipsychotic medication, regardless of dosage, for more than 4 weeks lifetime; (7) No known neurological- or endocrine disorders; (8) No mental retardation; (9) Understand and speak Norwegian; (10) Able to understand and sign an informed consent form. Written informed consent was obtained from study participants. Parents or legal guardians gave informed consent for patients younger than 16 years of age, as in Norway patients are legally able to consent without parental approval from the age of 16. The study was approved by the Regional Committee for Medical Research Ethics Sør-Øst C [REK Sør-Øst C (ref. no. 2009/949)].

### Clinical Measures

The Structural Interview for Prodromal Syndromes (SIPS) (Miller et al., 1999, 2003) was used for defining the UHR state. For this study the Norwegian translation of the SIPS version 5.0 was used (McGlashan et al., 2012). Further, the UHR subjects were screened diagnostically using the Clinical Interview for DSM-IV Axis Disorders (SCID) (First et al., 1994). The healthy controls went through a screening process with The Mini-International Neuropsychiatric Interview (MINI) (Sheehan et al., 2009) and Positive- and Negative Syndrome Scale (PANSS) (Kay et al., 1987) to exclude mental disorders. A screening assessment package which in addition to dichotic listening included tests measuring working memory, psychomotor speed, memory and learning, language and general knowledge and visuospatial abilities was also administered. Handedness was self-reported.

### Assessment Procedure

Psychiatric nurses trained in interviewing for psychosis spectrum disorders conducted the SIPS interviews. The SCID interviews were conducted by clinical psychologists. Consensus regarding the UHR state was reached during weekly diagnostic meetings.

### Dichotic Listening Procedure

The Bergen dichotic listening paradigm as described in Hugdahl and Andersson (1986), Hugdahl et al. (2009b), and Løberg et al. (2015) consists of auditory stimuli combining pairs of one consonant and the vowel a (ba, da, ga, ka, pa, ta, ka). Thirty-six pairs of syllables were presented during three conditions (non-forced, forced-right, forced-left). All syllables were spoken by a male voice with constant intensity, intonation and a neutral emotional connotation. The subjects were instructed to respond to the syllable they heard the best by pointing to an A4 sheet where the six different syllables were written in bold letters. Five trials were administered to ensure the subjects understood the task. In the forced conditions an arrow was placed in front of the subjects as a reminder of which ear to attend to. The pair of syllables was presented through a MP3 player (Sony-NWZ-E463) at an intensity which was adjusted individually to a comfortable level for each subject. In the non-forced condition two syllables were presented simultaneously, one in each ear. The subjects were instructed to report the syllable they heard the best. In the forced-left condition the subjects were asked to report the syllable from the left ear and in the forced-right condition the subjects were asked to report the syllable from the right ear. The syllables were temporally aligned to achieve simultaneous onset. The stimulus duration varied between 400 and 450 ms, and the inter-trial interval was 4 s. To avoid carry-over effects the presentation of forced-left and forced-right conditions were pseudorandomized and counterbalanced in the order ABBABA.

### Data Analysis

The statistical analyses were conducted using IBM Corp., IBM SPSS statistic version 22.0 (Statistical Package for the Social Sciences). Three laterality indices for language lateralization, one for each condition of dichotic listening, were calculated (Asbjørnsen and Bryden, 1996; Løberg et al., 2015) according to the following formula: [(right ear score) - (left ear score))/((right ear score) + (left ear score)]^∗^100. A subject with a positive laterality index is reporting more syllables from the right ear than from the left and a subject with a negative laterality index is reporting more syllables from the left ear than from the right. In order to quantify the effect of attention, two attentional gain scores were computed; (1) the increase in the number of correct right-ear reports from the non-forced to the forced-right condition (REgain = FR_RE – NF_RE); and (2) the increase of correct left-ear report from the non-forced to the forced-left condition (LEgain = FL_LE – NF_LE). All variables were visually inspected with regard to distribution using histograms and were deemed suitable for parametrical statistical analyses. Language lateralization and attention were analyzed using a three-way split-plot repeated measures analysis of covariance (ANCOVA) with group (UHR subjects vs. controls) as between-subjects factor and attention conditions (non-forced, forced-right, forced-left) and side (right ear, left ear) as repeated measures (2×3×2 design). Gender, age and handedness were used as covariates.

In the UHR subjects, relationships between SIPS subscales (Positive Symptoms, Negative Symptoms, Disorganized Symptoms, General Symptoms) and measures of attention and language lateralization derived from dichotic listening (laterality indices right- and left ear gain) were analyzed using Spearman’s correlation coefficients. Correlations between the items “Perceptual Abnormalities/Hallucinations” (P4), “Disorganized Communication” (P5). “Expression of Emotions” (N3), “Trouble with Focus and Attention” (D3) and dichotic listening performance (right- and left-ear gain, laterality indices) were also conducted. A Bonferroni corrected alpha level of 0.05/24 (0.002) was applied to assess statistical significance in these analyses.

## Results

**FIGURE 1:**
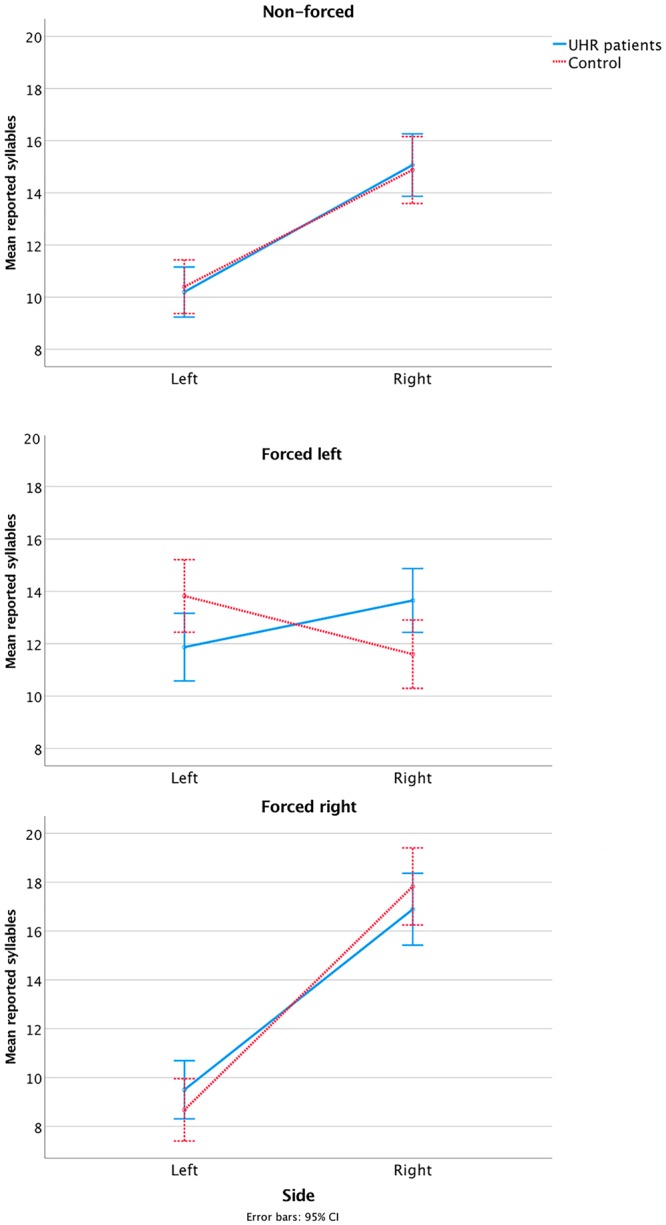
Mean reported syllables from each ear during the three dichotic listening conditions from the UHR patients and the healthy controls (error bars: 95% CI).

Forty-six UHR subjects and 40 healthy controls were included in this study. The demographics from the UHR subjects and healthy controls are described in **Table 1**. Mauchly’s test of sphericity indicated that the assumption of sphericity had been violated for the three way interaction, X2 (2) = 42.4, *p* < 0.05. Thus, the degrees of freedom were corrected using the Huynh–Feldt estimate of sphericity (𝜀 = 0.753). The three-way split-plot repeated measures ANCOVA revealed no main effects of group, side or condition. However, there was a statistically significant three-way interaction of group × condition × side, *F* = (2,81) = 3.438, *p* = 0.048. This analysis is illustrated in **Figure 1** which clearly shows that there is an interaction-effect in forced-left condition, but not in the non-forced or forced right conditions. Follow-up analyses of the three-way interaction using simple two-way interaction analyses for each condition showed that there was an unadjusted statistically significant interaction between group × side in the forced-left condition *F* = (1,81) = 4.466, *p* = 0.038, but no significant other interactions. The Bonferroni-corrected alpha level given three follow-up tests would be 0.016.

**Table 1:** Demographics and clinical characteristics by group (*n* = 86).

	Ultra high risk (*n* = 46)	Healthy controls (*n* = 40)
Age (SD)	17.87 (4.92)	16.80 (2.96)
Gender (female/male)	28/18	20/20
Handedness (right/left)	41/5	35/5
Positive symptoms (SIPS)	10.04 (3.77)	na^∗^
Negative symptoms (SIPS)	11.10 (6.67)	na^∗^
Disorganization symptoms (SIPS)	3.31 (2.58)	na^∗^
General symptoms (SIPS)	8.79 (3.63)	na^∗^

*^∗^na, not applicable.*

### UHR Symptoms and Dichotic Listening Performance

The SIPS item “Perceptual Abnormalities/Hallucinations” (P4) correlated positively with right ear gain (*r* = 0.486, *p* = 0.001) and was the only significant association when applying the Bonferroni-corrected alpha level (0.002). For exploratory purposes we hereby also report significant findings according to an alpha level of 0.05. The “Positive Symptoms” subscale correlated positively with right ear gain (*r* = 0.309, *p* = 0.036). The “Disorganized Symptoms” subscale correlated negatively with both left ear gain (*r* = -0.379, *p* = 0.009) and with the non-forced laterality index (*r* = -0.299, *p* = 0.044). “Trouble with Focus and Attention” (D3) correlated negatively with left ear gain (*r* = -0.334, *p* = 0.023). For detailed information regarding score distribution on SIPS refer to **Table 2**.

**Table 2:** Distribution of items from the SIPS interview (*n* = 46).

SIPS item score	0	1	2	3	4	5	6
“Perceptual abnormalities/hallucinations” (P4)	4	0	5	8	18	11	0
“Disorganized communication” (P5)	20	3	9	12	2	0	0
“Expression of emotion” (N3)	25	2	5	11	2	1	0
“Trouble with focus and attention” (D3)	11	2	6	18	9	0	0

## Discussion

To our knowledge this is the first study to assess language lateralization and attention mechanisms in UHR subjects using a dichotic listening test. We did not find support for Hypothesis 2 concerning reduced language lateralization in UHR, as performance in the non-forced condition was similar in UHR and controls. However, Hypothesis 1 was supported as the UHR subjects demonstrated difficulties in the condition where they were instructed to report syllables from the left ear (forced-left). This may be related to difficulties in applying top-down attention to override the bottom-up driven salience which arises when verbal stimuli are presented to the right ear. The forced-left condition has been labeled conflict resolution as the task requires suppression of a more salient stimulus (right ear) in order to report a less salient stimulus (left ear).

Further, right ear gain did correlate with the SIPS P4 item which measures hallucinations and perceptual abnormalities. Right ear gain is a measure of top-down attention- how much the subjects gain when they are instructed to report the most salient stimuli from the right in the forced-right condition as compared to when instructed to report the stimuli they hear most clearly in the non-forced condition.

The lack of findings regarding attenuated language asymmetry in UHR is not incommensurable with the literature on schizophrenia. Some studies indicate language asymmetry in patients with schizophrenia, shown as diminished right-ear advantage in the non-forced condition (Løberg et al., 1999; Ocklenburg et al., 2013). In other studies there was no difference between patients and controls (Løberg et al., 2002, 2015). In the present study **Figure 1** clearly illustrates that the UHR group and the healthy controls showed similar REAs in the non-forced condition. We expected to find indicators of mild language lateralization abnormalities in the UHR subjects, but dichotic listening thus failed to show any lateralization abnormalities. The inconsistencies in the literature and our lack of significant results may partly be resolved by the meta-analysis by Ocklenburg et al. (2013). This study quantified results from the non-forced condition across dichotic listening studies in patients with schizophrenia. They found that language lateralization abnormalities were weak in patients without auditory hallucinations (*g* = -0.26). When comparing schizophrenia patients with auditory hallucinations to healthy controls, the effect size was substantially stronger (*g* = -0.45). A possible explanation for the lack of language lateralization abnormalities in the UHR group in the present study could be that language laterality abnormalities appear in later stages of the psychosis continuum.

The present study found reduced report of left ear syllables in the forced left condition for the UHR group. This can be interpreted as a deficit in cognitive control. Cognitive control is a facet of goal directed behavior and mental flexibility, and implies an ability to allocate resources and prioritize information. It is the ability to maintain focus on designated stimuli when instructed to and not to be distracted by salient competing stimuli (Mackie et al., 2013). Healthy controls report more consonant-vowel syllables from the right ear in the non-forced condition, and more consonant-vowel syllables from the right ear in the forced-right condition. In the forced-left condition healthy controls are able to switch their attention away from the most salient stimuli from the right ear and thus demonstrate a left ear advantage (Asbjornsen and Hugdahl, 1995; Hugdahl et al., 2003). Patients with schizophrenia, however, often fail when there is a demand for attention in order to modulate stimulus driven right ear salience in the forced-left condition (Hugdahl et al., 2009b). Wang et al., 2005 compared the efficiency of three attentional networks (alerting, orienting, executive control) in schizophrenia, and the patients demonstrated deficits in attentional mechanisms of executive control and in the orienting network. In the present study, UHR subjects showed deficits in executive control in the forced-left condition. This is in line with studies of patients with schizophrenia which also demonstrate deficits in the forced-left condition when there is a cognitive conflicting situation (Nygård et al., 2013). Hugdahl et al. (2003) compared schizophrenia patients, patients with depression and healthy controls. They found that patients with schizophrenia were impaired in the forced-left condition. Both the healthy controls and patients with depression were able to direct their attention to the less salient stimuli from the left when instructed to. This illustrate that the impairment in top-down attentional mechanisms were distinct for patients with schizophrenia compared to subjects with another psychiatric disorder. Our findings are in accordance with the findings concerning the schizophrenia group. The present study revealed similar impairments in top-down attentional mechanisms in the forced-left condition in the UHR group as previously found in patients with schizophrenia, and this indicates that impaired auditory attentional mechanisms in the UHR state and schizophrenia may be comparable.

Studies with dichotic listening in schizophrenia have investigated positive symptoms (Løberg et al., 2006) and investigated the P3 item from PANSS called “Hallucinatory behavior” (Løberg et al., 2006, 2015; Hugdahl et al., 2013). “Perceptual abnormalities/hallucinations” (P4) from SIPS include questions about perceptual distortions, hallucinations, illusions of auditory, visual and somatic character. Our original assumption was that higher P4 scores in SIPS would be associated with deficits in attentional mechanisms. Hugdahl et al. (2013) showed that the more pronounced auditory hallucinations were, the less the patients were able to direct their attention to both the right ear syllable in the forced-right condition and the left ear in the forced-left condition. In the present study, the UHR subjects showed no impairment in directing their focus to the right ear in the forced-right condition. A direct comparison between these studies should be interpreted with caution because the UHR subjects do not report/experience persisting auditory hallucinations over the psychotic threshold. In the forced-right condition top-down and bottom-up mechanisms act synergistically due to the fact that both processes are pushing for a right ear response which cause an even stronger REA (Hugdahl, 2009). Our findings are novel and to our knowledge never reported elsewhere, thus the somewhat contraintuitive findings regarding perceptual abnormalities and hallucinations correlating positively with right ear gain in the UHR group must be replicated for any firm conclusions to be drawn, as the findings could be spurious.

A recent study (Glenthøj et al., 2017) found that negative symptoms were mediators between neurocognition and functional outcome. In the present study, negative symptoms were not related to neurocognition as measured by performance on dichotic listening. This is in accordance with a Structural Equation Model (SEM) presented in Løberg et al. (2006) where no significant differences emerged between dichotic listening performance and negative symptoms.

### Limitations and Suggestions for Further Research

Due to the novel findings not reported elsewhere, and sample size, conclusions drawn from SIPS derived symptoms and dichotic listening performance in UHR subjects in this study need to be replicated for any firm conclusion to be drawn. Future dichotic listening studies in the UHR population could also include a first-episode psychosis group and/or patients with schizophrenia, with and without auditory hallucinations to explore the continuum model of psychosis. Conversion rate from the UHR state to psychosis is highly heterogeneous with a variation from 13 to 45% at 2 years follow-up (Fusar-Poli et al., 2013), and diagnostic interviews also vary regarding criteria for inclusion. This contributes to challenges in comparing different UHR studies with each other. Research by Lin et al. (2015) indicated that at follow-up, 28% of the non-converters reported attenuated psychotic symptoms. Over the follow up period 68% of the non-converters experienced non-psychotic psychiatric disorders; mood disorder (49%), anxiety disorder (35%), substance abuse disorders (29%). Thus, the UHR state could be a predictor for psychiatric disorders in general. Further, while the dichotic listening paradigm used in the study is well established in research on schizophrenia, it has limitations concerning semantics and affective elements. See for instance Özkarar et al. (2008) for a discussion regarding how lateralized frontal hypoactivation may interact with semantic and affective phenomena. In the future, results obtained from dichotic listening and other neuropsychological tests, which demonstrate attentional deficits in the UHR population, could contribute to a better understanding of the UHR state and be a supplement in identification of UHR subjects. Further, increased knowledge regarding attention deficits in the UHR state might lead to more targeted interventions (e.g., cognitive remediation).

## Author Contributions

IA and KB wrote the first drafts for the paper and conducted the statistical analysis. IA, KB, and KK interpreted the results. The present study is a part of a larger study “Primary Prevention of Psychosis” and JJ, IJ, KB, JG outlined this study. All the authors provided detailed comments on the paper across several drafts and contributed in editing the final manuscript, and they were all available for theoretical discussions during the process.

## Conflict of Interest Statement

The authors declare that the research was conducted in the absence of any commercial or financial relationships that could be construed as a potential conflict of interest.
